# Factors associated with uptake of services to prevent mother-to-child transmission of HIV in a community cohort in rural Tanzania

**DOI:** 10.1136/sextrans-2014-051907

**Published:** 2015-06-04

**Authors:** Annabelle Gourlay, Alison Wringe, Jim Todd, Caoimhe Cawley, Denna Michael, Richard Machemba, Georges Reniers, Mark Urassa, Basia Zaba

**Affiliations:** 1 Faculty of Epidemiology and Population Health, London School of Hygiene & Tropical Medicine, London, UK; 2 National Institute for Medical Research, Mwanza, Tanzania

**Keywords:** AFRICA, HIV, PREGNANCY, ANTIRETROVIRAL THERAPY

## Abstract

**Objectives:**

This study aimed to identify factors associated with access to HIV care and antiretroviral (ARV) drugs for prevention of mother-to-child transmission (PMTCT) of HIV among HIV-positive pregnant women in a community cohort in rural Tanzania (Kisesa).

**Methods:**

Kisesa-resident women who tested HIV-positive during HIV serosurveillance and were pregnant (while HIV-positive) between 2005 and 2012 were eligible. Community cohort records were linked to PMTCT and HIV clinic data from four facilities (PMTCT programme implemented in 2009; referrals to city-based hospitals since 2005) to ascertain service use. Factors associated with access to HIV care and ARVs during pregnancy were analysed using logistic regression.

**Results:**

Overall, 24% of women accessed HIV care and 12% accessed ARVs during pregnancy (n=756 pregnancies to 420 women); these proportions increased over time. In multivariate analyses for 2005–2012, being married, prior voluntary counselling and testing, increasing age, increasing year of pregnancy and increasing duration of infection were independently associated with access to care and ARVs. Residence in roadside areas was an independent predictor of access to care but not ARVs. There was no evidence of an interaction with time period.

**Conclusions:**

Access to PMTCT services was low in this rural setting but improved markedly over time. There were fairly few sociodemographic differentials although support for young women and those without partners may be needed. Further decentralisation of HIV services to more remote areas, promotion of voluntary counselling and testing and implementation of Option B+ are likely to improve uptake and may bring women into care and treatment sooner after infection.

## Background

Services for prevention of mother-to-child transmission (PMTCT) of HIV have been scaled up rapidly amidst recent commitments to eliminating vertical transmission, particularly from sub-Saharan Africa where most paediatric HIV infections occur.^1^

PMTCT programmes, through the provision of antiretroviral (ARV) drugs to HIV-infected mothers and infants, can substantially reduce the risk of vertical HIV transmission from >40% to <5%.^2^ However, the availability of this intervention has not necessarily translated into service uptake. Coverage with PMTCT ARVs ranged from 13% to >95% across 21 sub-Saharan African countries in 2012, and reasons for poor uptake are complex, as barriers exist at multiple levels.^1^ ^3^ ^4^

Most quantitative studies investigating PMTCT outcomes are restricted to individuals enrolled in health services. However, it is important to characterise women who do not access HIV or antenatal clinics (ANCs) in order to design programmes to reach *all* women who need services. Clinic-based studies are also limited by data available in clinic records or self-reported in interviews, whereas unexplored factors such as duration of HIV infection, previous HIV testing and HIV status of partners, may also predict service use.

Analyses of population-level access to health services that link community-level data such as demographic surveillance systems (DSS) to health facility records are rarely undertaken. Studies in Malawi and Kenya reported population-level coverage of PMTCT or HIV services but did not investigate factors associated with service use.^5^ ^6^ Research in an Ugandan community-based cohort showed that distance from health services predicted not receiving an HIV test,^7^ but did not investigate factors associated with receiving ARV drugs. To our knowledge, no studies have investigated factors associated with enrolment into PMTCT programmes or uptake of ARVs among all HIV-positive pregnant women at a population level, despite the importance for optimising PMTCT programmes. Identifying local barriers to PMTCT uptake is also essential to tailor programmes to the context. We therefore aim to identify factors associated with accessing PMTCT services, including ARV drugs, among HIV-positive pregnant women in a community cohort in rural Tanzania.

## Methods

### Setting

**Figure 1:**
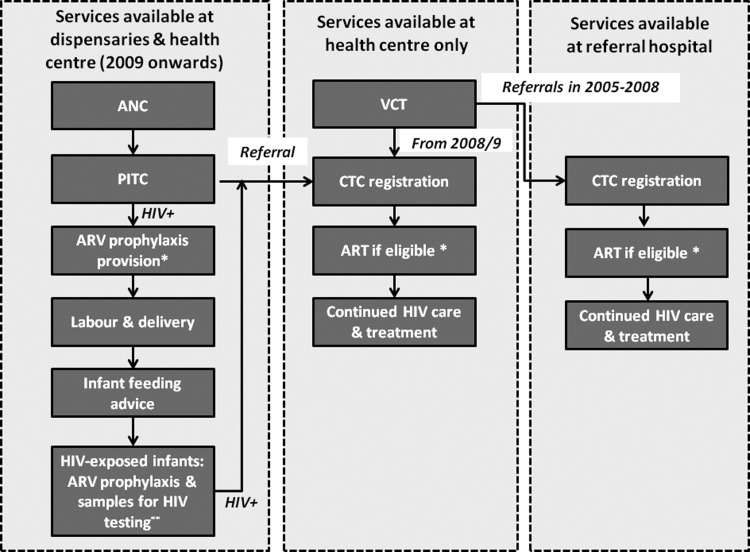
Cascade of prevention of mother-to-child transmission (PMTCT) services available in the dispensaries and/or health centre in Kisesa in 2009–2012, as well as referral services to city-based hospitals from 2005. ANC, antenatal clinic; PITC, provider-initiated testing and counselling; ARV, antiretroviral; CTC, care and treatment clinic; VCT, voluntary counselling and testing. From 2005 to 2009, pregnant women diagnosed with HIV at VCT services in the health centre were referred to hospitals in Mwanza city for PMTCT services. *In 2005–2011, HIV-positive pregnant women with CD4 counts <200 cells/mm^3^ were eligible for antiretroviral treatment (ART) for their own health (lamivudine (3TC) or emtricitabine, plus azidothymidine (AZT) or tenofovir, with efavirenz or nevirapine), otherwise ARV prophylaxis was provided: in 2005–2006 single-dose nevirapine at onset of labour; in 2007–2011 AZT from 28 weeks gestation (single-dose nevirapine, AZT and 3TC during labour) until 7 days postpartum (AZT plus 3TC). The treatment threshold was raised to 350 cells/mm^3^ in 2012 (ARV prophylaxis from 14 weeks (drug regimens remained unchanged), ‘Option A’).^30^ **In 2005–2006 infants received nevirapine within 72 h of birth; 2007–2011, infants received nevirapine for 1 week after birth and AZT for up to 4 weeks. Under ‘Option A’ in 2012 they received nevirapine prophylaxis until 1 week after cessation of breastfeeding (4–6 weeks if replacement feeding). Infant dried blood spot samples were sent for HIV testing to the national referral hospital in Mwanza city.

Kisesa is a rural community in north-western Tanzania, 20 km east of Mwanza city in Magu district. Approximately 30 000 individuals inhabit the DSS area of roughly 150 km^2^ that includes a trading centre and five other villages (roadside, or rural, where housing is fairly dispersed). There are three village dispensaries and a health centre in the trading centre (government-run). Implementation of PMTCT services started in 2009, including provider-initiated HIV testing and counselling and provision of ARV prophylaxis at ANCs, with referrals to the HIV care and treatment clinic (CTC) in the health centre for long-term care and ARV therapy (ART) (see figure 1 and see online supplement 1 for PMTCT protocols). From 2005 to 2009, pregnant women diagnosed with HIV at voluntary counselling and testing (VCT) services in the health centre were referred to hospitals in Mwanza city for PMTCT services. At the health centre, ANC, CTC and VCT services are carried out in separate buildings. Antenatal HIV prevalence in Magu district has declined from 10.7% in 2000 to 8.9% in 2008.^8^ ^9^

### Data collection

The Kisesa cohort study started in 1994,^10^ with DSS enumeration of the entire population every 6 months. Enumerators visit households to record all births, pregnancies, migrations and deaths. HIV serological surveys are conducted approximately every 3 years (seven to date, most recently in 2013) within each village among resident adults aged ≥15 years. Participants consent to give blood for HIV research testing without results disclosure, are offered VCT, and are interviewed about economic activities, childbearing, use of health services, and knowledge of HIV.

Routine clinic data were collected retrospectively from all four Kisesa facilities (approximately 10% of women in the seventh serosurvey attended ANC outside Kisesa). All records from 2005 to 2012 were abstracted from ANC pregnancy registers (some records (<10%) from different clinics and time periods were missing), PMTCT programme registers and the CTC (including patients who enrolled at city-based CTCs and transferred back to Kisesa). Data were double-entered, apart from CTC data.

Community cohort data were linked to clinic data sets by matching on personal attributes (eg, age, sex, village of residence and pregnancy dates), using an algorithm developed from a gold standard of ANC numbers captured from women's ANC cards during DSS round 27 (2012). The algorithm had a sensitivity of 70% and positive predictive value of 98% for matching ANC clinic records, with a similar algorithm used to match CTC data (algorithms were based on a similar approach to Kabudula *et al*).^11^ ^12^ PMTCT register records were linked to ANC and CTC records using ANC or CTC registration numbers, respectively.

Sources of pregnancy data were child birth dates in the DSS (linked to mothers), mothers’ self reports of pregnancies or births in the DSS or serosurveys, and clinic pregnancy records.

### Statistical analysis

Women residing in Kisesa in 2005–2012, testing HIV-positive during any serosurvey, and pregnant during this interval were eligible. HIV seroconversion dates were estimated using the midpoint between first positive and last negative test dates. Prevalent cases were assumed to have seroconverted 3 years prior to their first positive test date (based on average duration of infection for seroincident cases). The denominator comprised HIV-positive pregnancies, excluding pregnancy records that lacked serosurvey interview data within 5 years.

Two outcomes were assessed: (1) enrolled in a PMTCT programme and/or CTC (‘HIV care’) during or before pregnancy, and (2) accessed ARV drugs during pregnancy. Enrolment in HIV care was defined as linkage of a DSS record to a PMTCT or CTC clinic record. Dates of clinic registration were aligned with pregnancy dates to verify service access during each pregnancy. Maternal ARV access was defined as receipt of ARV drugs documented on any ANC visit in PMTCT registers, or a CTC record indicating initiation or continuation of ART during pregnancy (before the recorded or estimated delivery date). Data on infant ARVs or HIV diagnoses could not be linked to mothers’ records.

Explanatory variables were constructed using DSS data or serosurvey questions, taking information from the round closest to the pregnancy. Knowledge of HIV transmission was assessed by asking respondents to mention any modes of HIV transmission. ART knowledge was assessed using the number of correct answers to five true or false statements about ART (detailed in table 1). Responses to knowledge questions after the pregnancy date were distinguished, as knowledge was hypothesised to change as a result of attending the clinic. Death of a child was defined as any self-reported miscarriage, stillbirth or child death after birth. VCT use prior to pregnancy was based on attendance at VCT in an earlier serosurvey, or self-reported VCT use before the pregnancy date. HIV status of partners was determined using DSS line numbers of the spouse and spousal serosurvey HIV test data. Age was modelled as a continuous variable; all other quantitative variables were categorised.

**Table 1:** Characteristics of pregnancies (n=756) to HIV-positive women in Kisesa and proportions accessing HIV care/ARVs by factor

Factor	Category	Total number (%) of pregnancies		Number (%) in ‘HIV care’		Number (%) accessed ARVs	
Age	<20	36	4.8	0	0.0	0	0.0
	20–29	339	44.8	73	21.5	29	8.6
	30–39	347	45.9	97	28.0	53	15.3
	40+	34	4.5	10	29.4	6	17.6
Year	2005–6	167	22.1	3	1.8	2	1.2
of pregnancy	2007–8	209	27.6	21	10.0	11	5.3
	2009–10	183	24.2	66	36.1	23	12.6
	2011–2012	197	26.1	90	45.7	52	26.4
Residence area	Rural	371	49.1	70	18.9	44	11.9
	Roadside	202	26.7	61	30.2	23	11.4
	Trading Centre	183	24.2	49	26.8	21	11.5
Marital status	Married now	529	70.0	138	26.1	67	12.7
	Never married	69	9.1	10	14.5	3	4.3
	Married before	158	20.9	32	20.3	18	11.4
Education	At least P5	472	62.5	119	25.2	60	12.7
	P1–4	80	10.6	13	16.3	8	10.0
	No education	203	26.9	48	23.6	20	9.9
Religion	Catholic	311	41.9	80	25.7	37	11.9
	Other Christian	386	52.0	84	21.8	38	9.8
	Muslim	21	2.8	7	33.3	5	23.8
	Traditional	25	3.4	4	16.0	4	16.0
Ethnicity	Sukuma	688	91.1	167	24.3	83	12.1
	Other	67	8.9	13	19.4	5	7.5
(Personal) income	Farming or manual work	438	58.2	102	23.3	51	11.6
	Some business	208	27.6	48	23.1	28	13.5
	None	107	14.2	30	28.0	9	8.4
Gravidity	1	86	11.4	10	11.6	4	4.7
(pregnancy number)	2	125	16.5	24	19.2	10	8.0
	3	150	19.8	24	16.0	11	7.3
	4	130	17.2	36	27.7	15	11.5
	≥5	265	35.1	86	32.5	48	18.1
Any children died	No	401	54.3	86	21.4	39	9.7
	Yes	338	45.7	89	26.3	46	13.6
Duration of HIV	≤2 years	219	29.0	25	11.4	12	5.5
infection	>2–4 years	209	27.6	39	18.7	12	5.7
	>4 years	328	43.4	116	35.4	64	19.5
Prior VCT	No	512	67.7	86	16.8	36	7.0
	Yes	244	32.3	94	38.5	52	21.3
Knowledge of HIV	None	96	12.7	11	11.5	6	6.3
transmission	MTCT	35	4.6	12	34.3	6	17.1
	Other modes	314	41.5	87	27.7	46	14.6
	No prior report*	311	41.1	70	22.5	30	9.6
ART knowledge†	≤2 correct statements	119	15.7	36	30.3	17	14.3
	3 correct statements	88	11.6	26	29.5	11	12.5
	4–5 correct statements	87	11.5	42	48.3	26	29.9
	No prior report*	462	61.1	76	16.5	34	7.4
Relatives with or	No	493	66.7	107	21.7	47	9.5
died from HIV	Yes	246	33.3	68	27.6	39	15.9
Know someone	No	515	71.7	113	21.9	53	10.3
taking ART	Yes	203	28.3	64	31.5	34	16.7
Partner HIV status	Unknown‡	450	59.5	135	30.0	66	14.7
	Positive (had VCT)	10	1.3	0	0.0	0	0.0
	Positive (no VCT)	34	4.5	4	11.8	3	8.8
	Negative	50	6.6	3	6.0	3	6.0
	No spousal link	212	28.0	38	17.9	16	7.5

Missing values: education (1); religion (13); ethnicity (1); income (3); children died (17); relatives died (17); know someone on ART (38).

*No prior report: knowledge data point after pregnancy, or from an earlier serosurvey questionnaire lacking the same question.

†Statements: “Drugs can only slow down HIV illness not stop it”; “ART drugs are very dangerous and can kill people”; “ART drugs have to be used for life”; “ART drugs are available free of charge in Tanzania”; “Everyone who is infected with HIV needs drugs”.

‡Unknown to the study investigators.

ART, antiretroviral treatment; ARV, antiretroviral; MTCT, mother-to-child transmission; P1–4, primary level 1–4 years; P5, primary level 5 years; VCT, voluntary counselling and testing.

Descriptive analyses, followed by bivariate and multivariate logistic regression analyses (deemed appropriate given the short and homogeneous follow-up time per pregnancy) were performed using Stata V.12 (StataCorp LP, Texas, USA) to identify independent predictors of access to HIV care or ARVs. All factors associated with the outcome (p≤0.1) in bivariate analyses were assessed in multivariate models, using a forwards stepwise approach. Variables were retained if they significantly improved the model fit (p≤0.1, based on likelihood ratio tests). Clustering due to multiple pregnancies per woman was accounted for using random effects, checking for quadrature stability. Interactions with calendar year of pregnancy or age were assessed. For continuous variables, departure from linearity was assessed using likelihood ratio tests.

### Ethical considerations

Informed consent was obtained from all serosurvey participants. Data typists and managers received ethics training. Names of patients with HIV were not visible to data typists. Data sets were stored on password-restricted computer networks.

## Results

### Participants

Among 9692 women of childbearing age residing in Kisesa in 2005–2012 who had ever attended a serosurvey, 848 (8.7%) tested HIV-positive. Of these, 520 were pregnant between 2005 and 2012; 443 since (estimated) HIV seroconversion (n=810 pregnancies). Fifty-four pregnancy records lacking serosurvey data within 5 years were excluded, yielding 756 pregnancies for analysis (420 women) (see online supplement 2).

The pregnant women were 30 years old on average. Most were married (70%), educated to primary level (73%), Christian (94%) and from the Sukuma tribe (91%) (table 1). Half lived in remote rural villages. Women primarily earned money through farming (58%), and/or small businesses (28%). Few used VCT prior to pregnancy (32%). In 2005–2006, 167 HIV-positive women were pregnant; 209 in 2007–2008, 183 in 2009–2010 and 197 in 2011–2012.

### Factors associated with enrolment in HIV care in Kisesa

Overall 180 HIV-positive women accessed HIV care (24% of 756 HIV-positive pregnancies); a range from 2% in 2005–2006 (n=3 out of 167) to 46% in 2011–2012 (n=90 out of 197) (table 1). In crude analyses, access to HIV care increased with increasing age (p<0.001, table 2). No women aged <20 years accessed care. Year of pregnancy was strongly associated with accessing care, increasing sharply in 2009 (p<0.001). Compared with women from rural villages, those from roadside areas (OR 2.5 (95% CI 1.3 to 4.8)) or from the trading centre (OR 1.6 (95% CI 0.8 to 3.2)) were more likely to enrol in care. Unmarried women had lower odds of being in care than married women (p=0.09). VCT prior to pregnancy (OR 6.7 (95% CI 3.5 to 13.0)), higher gravidity (p<0.001) and increasing duration of HIV infection (p<0.001) were also associated with receiving care. Women who named HIV transmission modes (OR 5.3 (95% CI 1.2 to 24) for mother-to-child, OR 6.5 (95% CI 2.2 to 19) for other modes, vs none) and women who correctly answered ≥four ART statements (OR 4.5 (95% CI 1.6 to 13) vs ≤two correct statements), were more likely to access care. Having a relative with HIV (alive or dead) or knowing someone taking ART was associated with higher odds of being in care (OR 1.5 (95% CI 0.9 to 2.7) and OR 2.1 (95% CI 1.2 to 3.8) respectively). Women with HIV-positive or HIV-negative partners appeared less likely to access care compared with the larger group of women whose partners’ HIV status was unknown (had not attended serosurveys) (p<0.001). There was no statistical evidence for an association between enrolment in care and educational level, religion, ethnicity, source of income or death of a child. In multivariate analyses, increasing age, increasing year of pregnancy, residence in roadside areas, being married, increasing duration of infection and prior VCT were independently associated with greater access to care (table 2).

**Table 2:** Crude and multivariate logistic regression models for factors associated with access to HIV care and ARVs during pregnancy (n=756 pregnancies)

		Enrolled in HIV care						Accessed ARV drugs					
Factor	Category	OR	95% CI	p (LRT)	aOR	95% CI	p (LRT)	OR	95% CI	p (LRT)	aOR	95% CI	p (LRT)
Age*		1.2	1.1 to 1.3	<0.001	1.1*	1.0 to 1.2	0.04	1.1	1.1 to 1.2	<0.001	1.1	1.0 to 1.2	0.05
Year of pregnancy	2005–2006	1		<0.001	1		<0.001	1		<0.001	1		<0.001
	2007–2008	26.8	2.8 to 260		14	1.8 to 109		7.1	1.1 to 48		4.2	0.7 to 27	
	2009–2010	1753	75 to >5000		481	35 to >5000		415	5.1 to 325		21	3.0 to 142	
	2011–2012	7109	208 to >5000		1051	65 to >5000		207	21 to 2056		72	9.6 to 550	
Residence area	Rural	1		0.02	1		0.08	1		0.9			
	Roadside	2.5	1.3 to 4.8		3.4	1.0 to 11		1.0	0.5 to 2.0				
	Trading Centre	1.6	0.8 to 3.2		2.8	0.8 to 9.8		0.9	0.4 to 1.9				
Marital status	Married now	1		0.09	1		0.03	1		0.1	1		0.1
	Never married	0.3	0.1 to 1.0		0.2	0.0 to 1.1		0.3	0.1 to 1.1		0.2	0.0 to 1.3	
	Married before	0.8	0.4 to 1.5		0.3	0.1 to 1.0		1.0	0.5 to 1.9		0.7	0.3 to 1.6	
Education	At least P5	1		0.2				1		0.7			
	P1–4	0.4	0.2 to 1.2					0.7	0.3 to 1.9				
	no education	1.0	0.6 to 1.9					0.8	0.4 to 1.5				
Religion	Catholic	1		0.6				1		0.4			
	Other Christian	0.8	0.4 to 1.4					0.8	0.5 to 1.5				
	Muslim	1.5	0.3 to 7.1					2.6	0.6 to 11				
	Traditional	0.5	0.1 to 2.6					1.6	0.4 to 6.5				
Ethnicity	Sukuma	1		0.7				1		0.7			
	Other	0.8	0.3 to 2.2					0.8	0.3 to 2.2				
Personal income	Farming/manual	1		0.7				1		0.6			
income	Some business	1.0	0.5 to 1.9					1.1	0.6 to 2.2				
	None	1.4	0.6 to 3.0					0.7	0.3 to 1.7				
Gravidity (pregnancy number)	1	1		<0.001				1		<0.001			
	2	2.4	0.7 to 8.0					1.7	0.4 to 6.7				
	3	2.3	0.7 to 8.1					1.7	0.4 to 7.0				
	4	9.9	2.4 to 40					3.8	0.9 to 15				
	≥5	21.2	4.6 to 99					7.7	1.9 to 31				
Any children died	No	1		0.3				1		0.1			
died	Yes	1.4	0.8 to 2.3					1.6	0.9 to 2.8				
Duration of HIV infection	≤2 years	1		<0.001	1		0.002	1		<0.001	1		0.002
	>2–4 years	3.3	1.4 to 7.6		1.7	0.6 to 5.0		1.1	0.4 to 2.8		0.7	0.2 to 2.3	
	>4 years	20.7	6.9 to 61		7.4	2.1 to 25		6.4	2.7 to 15		3.7	1.2 to 11	
Prior VCT	No	1		<0.001	1		0.09	1		<0.001	1		0.02
	Yes	6.7	3.5 to 13		2.2	0.9 to 5.8		5.4	2.8 to 11		2.5	1.1 to 5.8	
Knowledge of HIV transmission	None	1		<0.001				1		0.02			
	MTCT	5.3	1.2 to 24					5.8	1.2 to 29				
	Other modes	6.5	2.2 to 19					3.5	1.1 to 11				
	No prior report†	2.6	1.0 to 7.3					1.7	0.6 to 5.1				
ART knowledge‡	≤2 correct	1		<0.001				1		<0.001			
	3 correct	0.9	0.3 to 2.5					1.1	0.3 to 3.3				
	4–5 correct	4.5	1.6 to 13					5.3	1.8 to 16				
	No prior report†	0.2	0.1 to 0.4					0.4	0.2 to 0.9				
Relatives with/died from HIV	No	1		0.1				1		0.02			
	Yes	1.5	0.9 to 2.7					2.0	1.1 to 3.6				
Know someone taking ART	No	1		0.01				1		0.02			
	Yes	2.1	1.2 to 3.8					2.0	1.1 to 3.7				
Partner HIV status	Unknown §	1		<0.001				1		0.02			
	Positive	0.2	0.0 to 0.6					0.3	0.1 to 1.4				
	Negative	0.1	0.0 to 0.4					0.3	0.1 to 1.1				
	No spousal link	0.4	0.2 to 0.8					0.5	0.2 to 0.9				

*Age modelled as a continuous variable, no evidence for departure from linearity (p=0.4 LRT).

†No prior report: knowledge data point after pregnancy, or from an earlier serosurvey questionnaire lacking the same question.

‡Statements: “Drugs can only slow down HIV illness not stop it”; “ART drugs are very dangerous and can kill people”; “ART drugs have to be used for life”; “ART drugs are available free of charge in Tanzania”; “Everyone who is infected with HIV needs drugs”.

§Unknown to the study investigators.

ART, antiretroviral treatment; ARV, antiretroviral; OR, OR (crude); aOR, adjusted OR; LRT, likelihood ratio test; MTCT, mother-to-child transmission; P1–5, primary level; VCT, voluntary counselling and testing.

### Factors associated with accessing ARV drugs

Eighty-eight women accessed ARV prophylaxis or ART during pregnancy (12% overall, 49% out of 180 in care). In bivariate analyses, factors associated with ARV access mirrored those associated with enrolment in care, except for area. Women whose children had died appeared more likely to access ARVs (p=0.1). Increasing age and year of pregnancy, being married, increasing duration of infection and prior VCT remained independently associated with improved ARV access in the final models.

### Factors over time

**Figure 2:**
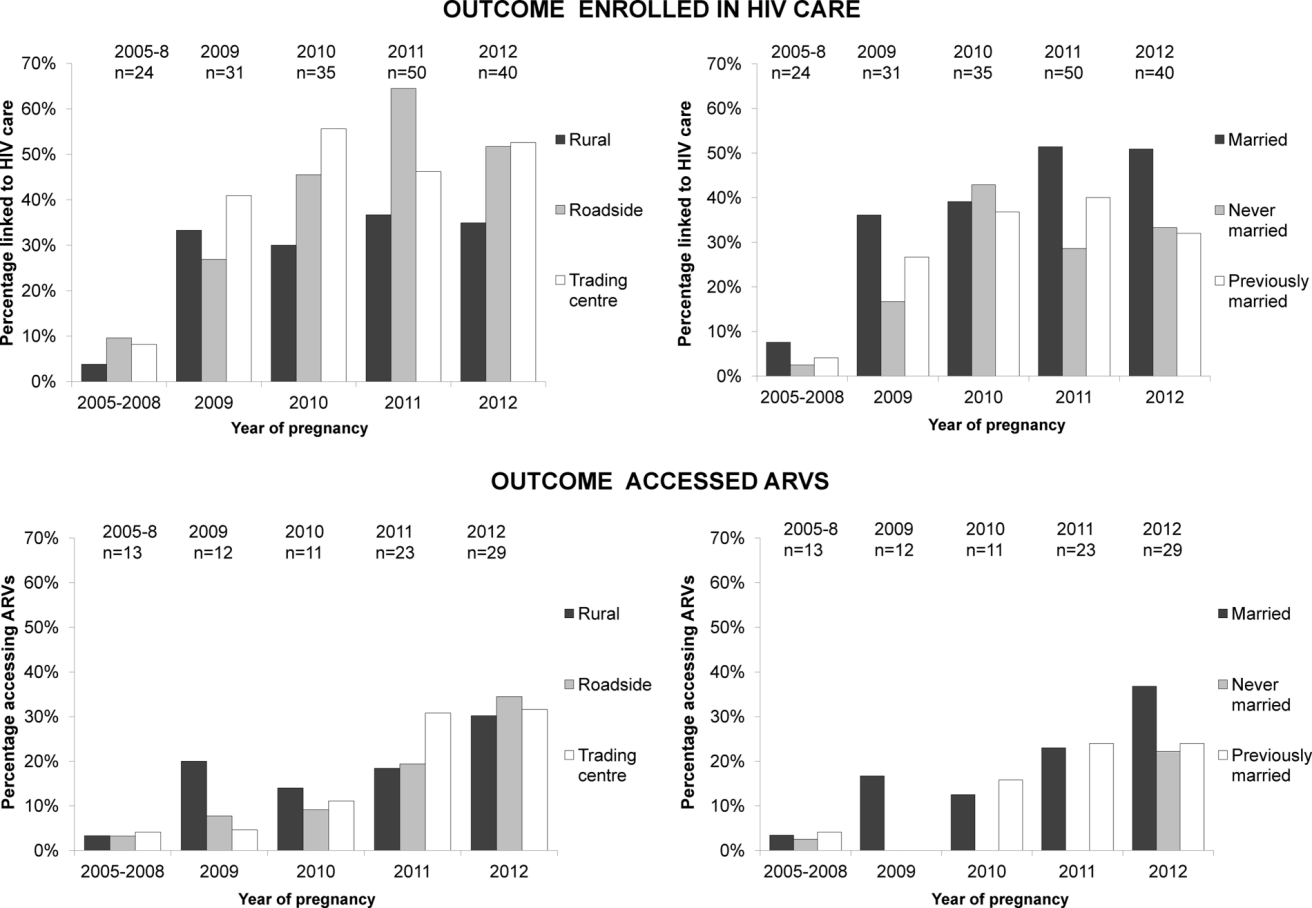
Proportion accessing HIV care by area or marital status, over time (top row); proportion accessing antiretrovirals (ARVs) during pregnancy by area or marital status over time (bottom row).

Patterns of access to care and ARVs by area or marital status were similar over time (figure 2). Rural women were generally disadvantaged, although the pattern was inverted in 2009–2010 for ARV usage. There was no statistical evidence for an interaction between year of pregnancy (or age) and area or other variables (Gourlay^11^ includes further details of uptake and coverage estimates over time).

Out of 50 women who had PMTCT in an earlier pregnancy, 42 (84%) accessed HIV care in their subsequent pregnancy.

### Comparison of linked and non-linked clinic records

Kisesa-resident ANC records that were not linked to the DSS (n=2579, 21%) did not differ in terms of age or year of ANC registration (marital status not recorded at ANC) to the linked ANC records (n=9842). However, compared to linked CTC records (n=661), a greater proportion of non-linked CTC records (n=174) were separated/widowed (19% vs 9%, p=0.003), or were registrations prior to 2008 (25% vs 3%, p<0.001) (data not shown), but there was no difference in age.

## Discussion

In this rural Tanzanian setting, access to PMTCT services was low but increased over time, with fairly few though sometimes strong inequities in service access. Area, marital status and age predicted service use, alongside previously unexplored factors including prior VCT and duration of HIV infection—findings that could not have been deduced from a clinical cohort. Year of pregnancy was, perhaps unsurprisingly, the strongest predictor of access to care and ARVs, the large effect sizes reflecting the availability of PMTCT services in Kisesa from 2009. The increasing complexity and duration of PMTCT drug regimens over time, as guidelines changed, did not appear to hinder access to PMTCT care and ARVs, although it may have had an impact on subsequent adherence which we were not able to measure.

Pregnant women living in rural areas were less likely to enrol in HIV care, with disparities persisting over time, likely reflecting the greater distance, time and cost of travel to health services; barriers that emerged in qualitative research on PMTCT in this setting.^13^ Poorer uptake of VCT in remote villages in Kisesa has also been documented.^14^ ^15^ Further decentralisation of CTC services to more rural areas, while maintaining regular supplies of HIV test kits and drugs (a major issue during the study, particularly in dispensaries),^13^ ^16^ will be important in ensuring all women in need of PMTCT services are reached. Surprisingly, area of residence was not associated with uptake of ARV drugs. This may be explained in part by the opposing patterns of access to ARVs by area when disaggregated by time period.

Unmarried HIV-positive pregnant women were less likely to access care and acquire ARV drugs than married women, with little change over time, potentially reflecting an absence of support from male partners—an important determinant of PMTCT service use in qualitative research in Kisesa and elsewhere.^4^ ^17^ In contrast, a few African studies found married or cohabiting women were less likely to use PMTCT ARVs or other HIV services,^14^ ^18^ ^19^ perhaps due to perceived negative reactions from partners. Support for HIV-positive women through NGOs, community-based organisations (eg, Mothers2mothers)^20^ or relatives may improve access.

Young pregnant women were particularly disadvantaged in this setting, mirroring findings from several African studies.^21–23^ Qualitative research in Kisesa and South Africa suggests that young HIV-positive pregnant women face discrimination in health facilities.^16^ ^21^ Providing additional support tailored to young HIV-positive pregnant women and improving health workers’ behaviour through training and supervision may encourage attendance.

Increasing duration of HIV infection was a strong predictor of access to HIV care in pregnancy, presumably reflecting development of symptoms driving individuals to seek care. Shortages of HIV-test kits and consequent prioritisation of testing and enrolment for symptomatic women may also explain this finding. Some women who accessed HIV care were already attending the CTC before pregnancy, and may have been referred from VCT services with symptoms. The association between infection duration and ARV uptake also reflects ART eligibility with increasing disease progression, although ineligible women should have been offered prophylaxis. Qualitative research in East Africa suggests that asymptomatic HIV-positive pregnant women feel ARVs for PMTCT are unnecessary,^24–26^ supporting our findings. Promoting VCT attendance, independently associated with access to HIV care and ARVs during pregnancy, may bring women into care and treatment earlier in their infection. Implementation of Option B+ (lifelong ART for all HIV-positive pregnant women) could also provide an incentive for pregnant women to seek care and treatment earlier.^27^

Unlike studies of VCT uptake in Kisesa,^14^ ^28^ we found no evidence that HIV knowledge or education predicted PMTCT service enrolment. However, our findings are consistent with recent quantitative research on PMTCT service use.^4^ Contrasting findings between HIV services may reflect differences in service models and motivations for clinic attendance, in which individuals seeking VCT actively decide to learn their status, compared with pregnant women seeking routine ANC services. Partner HIV status strongly predicted VCT uptake in Kisesa, but was not independently associated with access to PMTCT in this analysis. However, the small fraction of women linked to partners with test results limited the statistical power and conclusions we could draw.

The primary strength of this analysis was the linkage of community cohort and clinic data, enabling a population-level investigation of factors associated with PMTCT service use. Nevertheless, we were unable to link some clinic records to the DSS (adjusted coverage estimates are presented elsewhere^29^). Unlinked clinic records did not differ in age, but were more commonly separated/widowed women, potentially biasing our estimates for marital status. Fewer clinic records from earlier time periods were linked, so the association with year of pregnancy may be overestimated, although this is unlikely to have affected our conclusions. Selection bias is conceivable as serosurvey attenders might be in worse health than non-attenders (free treatment for non-HIV conditions is provided). Estimated HIV seroconversion dates may be inaccurate, although a sensitivity analysis limited to seroincident cases revealed the same associations. The sample size limited the power to investigate factors by time period, and was possibly too small to detect smaller effects in multivariate analyses. We were unable to investigate later PMTCT service outcomes, due to data quality issues that hindered linkage of delivery or postpartum records, or to examine ARV drug adherence. The true benefit of services may therefore be overestimated, although factors associated with later outcomes would likely be similar. A fraction of women may have accessed ANC outside Kisesa and been misclassified as non-attenders (we were unable to collect ANC records from clinics outside the area for cost and logistical reasons). While the set of factors associated with service use will vary by location, our findings may inform PMTCT programmes elsewhere in Tanzania and rural Africa.

In conclusion, access to PMTCT services was low but improved substantially over time, with a few strong sociodemographic differentials. Additional support for young or unmarried women may be needed. Programmatic factors such as accessibility, HIV-test kit and drug availability, previous HIV service use and patient-provider interactions may be strong drivers of service use in this setting. Further decentralisation and strengthening of PMTCT services is necessary, while promoting VCT and implementing Option B+ may bring women into care and treatment sooner after HIV infection.

**Key messages**

- The majority of HIV-positive pregnant women did not access available prevention of mother-to-child transmission (PMTCT) services in this rural African setting.
- Limited service accessibility remains an issue for women living in more remote areas, necessitating further decentralisation of HIV and PMTCT services.
- Additional support is needed for young pregnant HIV-positive women and those without partners.
- Promoting use of voluntary counselling and testing services and implementing Option B+ may bring HIV-positive women into care at an earlier stage of HIV infection.

## Supplementary Material

Web figure 1

Web figure 2

## References

[1] UNAIDS. The Gap Report. Geneva, Switzerland, 2014.

[2] WHO. PMTCT Strategic Vision 2010–2015: preventing mother-to-child transmission of HIV to reach the UNGASS and Millennium Development Goals. World Health Organization, 2010.

[3] UNAIDS. Global Report: UNAIDS report on the global AIDS epidemic 2013. Geneva, Switzerland, 2013.

[4] Gourlay A, Birdthistle I, Mburu G, et al Barriers and facilitating factors to the uptake of antiretroviral drugs for prevention of mother-to-child transmission of HIV in sub-Saharan Africa: a systematic review. J Int AIDS Soc 2013;16:18588 10.7448/IAS.16.1.18588 23870277 PMC3717402

[5] Price AJ, Kayange M, Zaba B, et al Uptake of prevention of mother-to-child-transmission using Option B+ in northern rural Malawi: a retrospective cohort study. Sex Transm Infect 2014;90:309–14. 10.1136/sextrans-2013-051336 24714445 PMC4033143

[6] Gargano JW, Laserson K, Muttai H, et al The adult population impact of HIV care and antiretroviral therapy in a resource poor setting, 2003–2008. AIDS 2012;26:1545–54. 10.1097/QAD.0b013e328353b7b9 22441254

[7] Larsson EC, Thorson AE, Pariyo G, et al Missed Opportunities: barriers to HIV testing during pregnancy from a population based cohort study in rural Uganda. PLoS One 2012;7:e37590 10.1371/journal.pone.0037590 22916089 PMC3420922

[8] Keogh SC, Urassa M, Kumogola Y, et al Reproductive behaviour and HIV status of antenatal clients in northern Tanzania: opportunities for family planning and preventing mother-to-child transmission integration. AIDS 2009;23 (Suppl 1):S27–35. 10.1097/01.aids.0000363775.68505.f1 20081386

[9] Urassa M, Kumogola Y, Isingo R, et al HIV prevalence and sexual behaviour changes measured in an antenatal clinic setting in northern Tanzania. Sex Transm Infect 2006;82:301–6. 10.1136/sti.2005.016766 16877579 PMC2564714

[10] Mwaluko G, Urassa M, Isingo R, et al Trends in HIV and sexual behaviour in a longitudinal study in a rural population in Tanzania, 1994–2000. AIDS 2003;17:2645–51. 10.1097/00002030-200312050-00012 14685059

[11] Gourlay A Improving the usage of prevention of mother-to-child transmission of HIV services in rural Tanzania. London School of Hygiene and Tropical Medicine, 2015.

[12] Kabudula CW, Clark BD, Gomez-Olive FX, et al The promise of record linkage for assessing the uptake of health services in resource constrained settings: a pilot study from South Africa. BMC Med Res Methodol 2014;14:71 10.1186/1471-2288-14-71 24884457 PMC4041350

[13] Gourlay A, Mshana G, Wringe A, et al Barriers to uptake of prevention of mother-to-child transmission of HIV services in rural Tanzania: a qualitative study. Global Maternal Health Conference 2013 Arusha, Tanzania, 2013.

[14] Wringe A, Isingo R, Urassa M, et al Uptake of HIV voluntary counselling and testing services in rural Tanzania: implications for effective HIV prevention and equitable access to treatment. Trop Med Int Health 2008;13:319–27. 10.1111/j.1365-3156.2008.02005.x 18397395

[15] Isingo R, Wringe A, Todd J, et al Trends in the uptake of voluntary counselling and testing for HIV in rural Tanzania in the context of the scale up of antiretroviral therapy. Trop Med Int Health 2012;17:e15–25. 10.1111/j.1365-3156.2011.02877.x 22943375 PMC3443372

[16] Gourlay A, Wringe A, Birdthistle I, et al “It Is Like That, We Didn't Understand Each Other”: exploring the influence of patient-provider interactions on prevention of mother-to-child transmission of HIV service use in rural Tanzania. PLoS One 2014;9:e106325 10.1371/journal.pone.0106325 25180575 PMC4152246

[17] Gourlay A, Birdthistle I, Wringe A, et al Challenges with male involvement in prevention of mother-to-child transmission HIV services in rural Tanzania: views of fathers, mothers and providers AIDS. Impact Conference, Barcelona, 2013.

[18] Ekouevi DK, Leroy V, Viho A, et al Acceptability and uptake of a package to prevent mother-to-child transmission using rapid HIV testing in Abidjan, Cote d'Ivoire. AIDS 2004;18:697–700. 10.1097/00002030-200403050-00018 15090779

[19] Muchedzi A, Chandisarewa W, Keatinge J, et al Factors associated with access to HIV care and treatment in a prevention of mother to child transmission programme in urban Zimbabwe. J Int AIDS Soc 2010;13:38 10.1186/1758-2652-13-38 20925943 PMC2978127

[20] Baek C, Mathambo V, Mkhize S, et al Key Findings from an Evaluation of the mothers2mothers Program in KwaZulu-Natal, South Africa. Horizons Final Report Washington DC, 2007.

[21] Varga C, Brookes H Factors influencing teen mothers’ enrollment and participation in prevention of mother-to-child HIV transmission services in Limpopo Province, South Africa. Qual Health Res 2008;18:786–802. 10.1177/1049732308318449 18503020

[22] Kirsten I, Sewangi J, Kunz A, et al Adherence to combination prophylaxis for prevention of mother-to-child-transmission of HIV in Tanzania. PLoS One 2011;6:e21020 10.1371/journal.pone.0021020 21695214 PMC3112206

[23] Stringer EM, Ekouevi DK, Coetzee D, et al Coverage of nevirapine-based services to prevent mother-to-child HIV transmission in 4 African countries. JAMA 2010;304:293–302. 10.1001/jama.2010.990 20639563

[24] Duff P, Kipp W, Wild TC, et al Barriers to accessing highly active antiretroviral therapy by HIV-positive women attending an antenatal clinic in a regional hospital in western Uganda. J Int AIDS Soc 2010;13:37 10.1186/1758-2652-13-37 20863399 PMC2954932

[25] Levy JM Women's expectations of treatment and care after an antenatal HIV diagnosis in Lilongwe, Malawi. Reprod Health Matters 2009;17:152–61. 10.1016/S0968-8080(09)33436-9 19523592

[26] Theilgaard ZP, Katzenstein TL, Chiduo MG, et al Addressing the fear and consequences of stigmatization—a necessary step towards making HAART accessible to women in Tanzania: a qualitative study. AIDS Res Ther 2011;8:28 10.1186/1742-6405-8-28 21810224 PMC3173282

[27] WHO. Consolidated guidelines on the use of antiretroviral drugs for treating and preventing HIV infection. Geneva, Switzerland, 2013. 24716260

[28] South A, Wringe A, Kumogola Y, et al Do accurate HIV and antiretroviral therapy knowledge, and previous testing experiences increase the uptake of HIV voluntary counselling and testing? Results from a cohort study in rural Tanzania. BMC Public Health 2013;13:802 10.1186/1471-2458-13-802 24007326 PMC3844310

[29] Gourlay A, Wringe A, Todd J, et al Uptake of services for prevention of mother-to-child transmission of HIV in a community cohort in rural Tanzania from 2005 to 2012. Submitted to JAIDS 2014. In press. 10.1186/s12913-015-1249-6 PMC4702391 26739028

[30] Ministry of Health and Social Welfare. National Guidelines for Comprehensive Care of Prevention of Mother-to-Child Transmission HIV Services. Tanzania, 2012.

