# Supplementary material for: Factors associated with uptake of services to prevent mother-to-child transmission of HIV in a community cohort in rural Tanzania
Source: Sex Transm Infect. 2015 Jun 4;91(7):520–7. doi: 10.1136/sextrans-2014-051907 (PMC4680170; doi:10.1136/sextrans-2014-051907)
Supplement: Web figure 1 [file sextrans-2014-051907-s1.pdf]

Supplementary figure 1. Timeline of PMTCT guideline and services in Kisesa

PMTCT guidelines and regimen changes	Year	Implementation of services in Kisesa
ART if CD4<200<br>Single-dose nevirapine during labour and to infant within 72 hours →	2005	← VCT clinic opens in Kisesa health centre (HIV-positive pregnant women are referred to Mwanza city hospitals)
	2006	
AZT from 28 weeks gestation until 7 days postpartum*, infants receive nevirapine for 1 week/ AZT for up to 4 weeks →	2007	
	2008	
	2009	← CTC opens in Kisesa health centre<br>← PMTCT services start in Kisesa health centre and dispensaries
	2010	
	2011	
ART if CD4<350<br>Option 'A': AZT from 14 weeks gestation until 7 days postpartum*, infants receive nevirapine until 1 week after breastfeeding cessation →	2012	

*single-dose nevirapine + azidothymidine(AZT) + lamivudine during labour; AZT+lamivudine postpartum
