# Supplementary material for: Factors associated with uptake of services to prevent mother-to-child transmission of HIV in a community cohort in rural Tanzania
Source: Sex Transm Infect. 2015 Jun 4;91(7):520–7. doi: 10.1136/sextrans-2014-051907 (PMC4680170; doi:10.1136/sextrans-2014-051907)

Flow chart of number of participants and pregnancies included in the analysis and flow through the PMTCT cascade

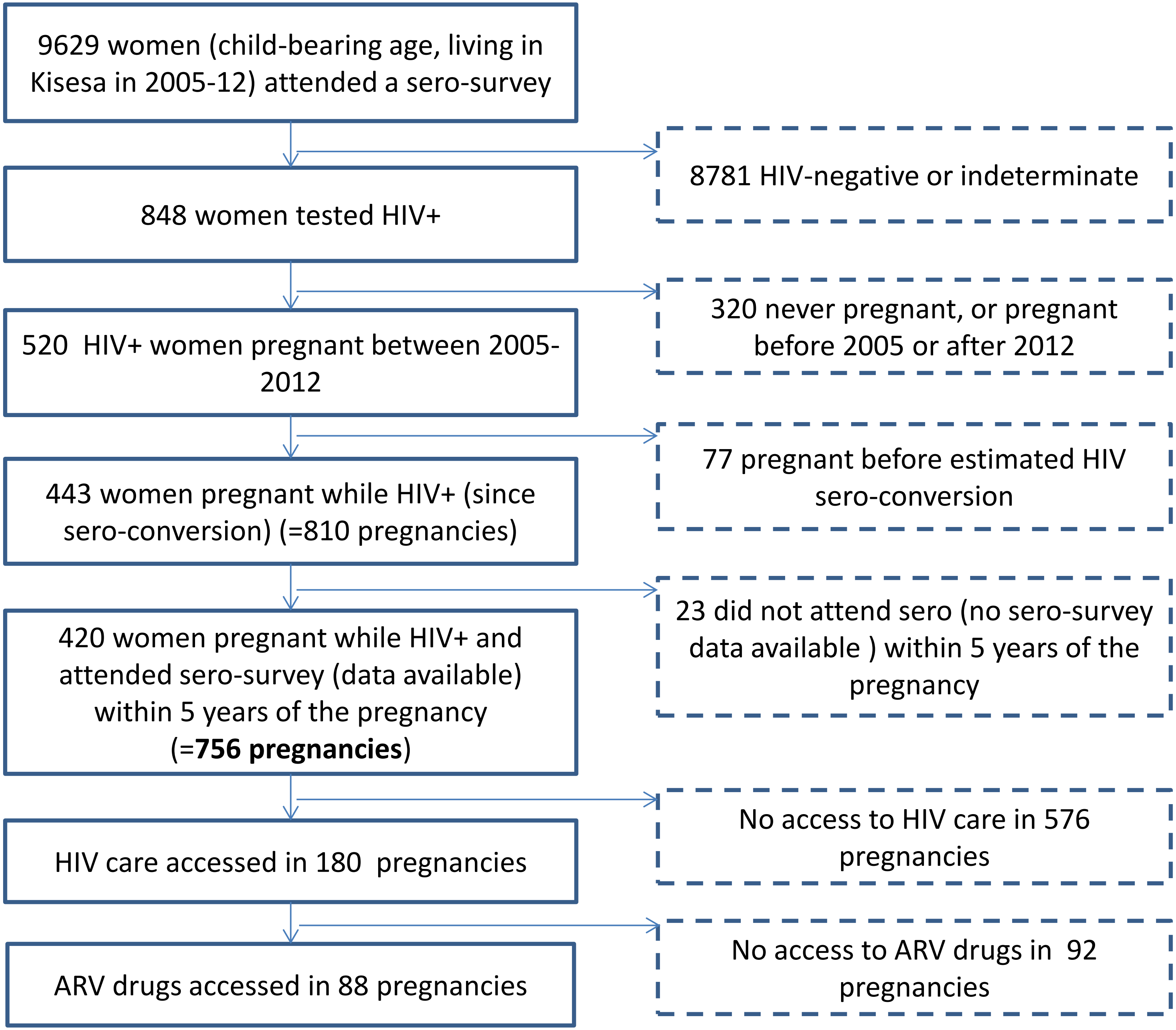

Supplement: Web figure 2 [file sextrans-2014-051907-s2.pdf]
